# *SYNE1‐QK1* SNPs, G × G and G × E interactions on the risk of hyperlipidaemia

**DOI:** 10.1111/jcmm.15239

**Published:** 2020-04-13

**Authors:** Peng‐Fei Zheng, Rui‐Xing Yin, Chun‐Xiao Liu, Guo‐Xiong Deng, Yao‐Zong Guan, Bi‐Liu Wei

**Affiliations:** ^1^ Department of Cardiology Institute of Cardiovascular Diseases The First Affiliated Hospital Guangxi Medical University Nanning China; ^2^ Guangxi Key Laboratory Base of Precision Medicine in Cardio‐cerebrovascular Disease Control and Prevention Nanning China; ^3^ Guangxi Clinical Research Center for Cardio‐cerebrovascular Diseases Nanning China

**Keywords:** environmental factor, haplotype, hyperlipidaemia, interaction, KH domain containing RNA binding gene, single nucleotide polymorphism, spectrin repeat containing nuclear envelope protein 1

## Abstract

This study aimed to assess the relationship of 3 spectrin repeat containing nuclear envelope protein 1 (*SYNE1*) and 4 KH domain containing RNA binding (*QK1*) single nucleotide polymorphisms (SNPs), their haplotypes, gene‐gene (G × G), gene‐environment (G × E) interactions and hypercholesterolaemia (HCH) and hypertriglyceridaemia (HTG) in the Chinese Maonan minority. The genetic make‐up of the *SYNE1‐QK1* SNPs in 1932 unrelated subjects (normal, 641; HCH, 649; and HTG, 642) was obtained by next‐generation sequencing technologies. The genotypic frequencies of following SNPs were suggestively distinctive between the control and HCH groups (rs2623963, rs7745725, rs9459317, rs16897566), or between the control and HTG groups (rs2623963, rs1358317, rs7745725, rs1923608, rs16897566 SNPs; *P* < .05, respectively). Multiple‐locus linkage disequilibrium analysis indicated that the identified SNPs were not inherited independently. Several haplotypes and gene‐gene interaction haplotypes among the detected SNPs may be related with an increased morbidity of HCH (C‐G‐A, C‐G‐G and C‐G‐G‐T‐C‐A‐T) and HTG (C‐G‐G, G‐T‐G‐C, C‐G‐G‐G‐T‐G‐C and C‐G‐G‐T‐C‐A‐T), whereas others may be related with an decreased risk of HCH (G‐A‐A, G‐C‐A‐T, C‐A‐A‐T‐C‐A‐T and G‐A‐A‐G‐C‐A‐T) and HTG (G‐A‐A, G‐C‐A‐T, C‐A‐A‐T‐C‐A‐T and G‐A‐A‐G‐C‐A‐T). The association evaluation based on haplotypes and gene‐gene interactions could improve the power of detecting the risk of dyslipidaemia than anyone of SNP alone. There was significant three‐locus model involving SNP‐SNP, haplotype‐haplotype/environment and G × G interactions (*P* < .05‐0.001) that were detected by GMDR in HCH and HTG groups. Different interactions between genetic and environmental factors would produce different redundancy or synergy effects on the morbidity of HCH and/or HTG.

## 1 INTRODUCTION

Coronary artery disease (CAD) has become the prominent reason of morbidity, disability, functional deterioration, mortality and costly health care, plus it is responsible for around 30% of all the deaths globally.^1^, ^2^, ^3^ Preceding researches have showed that hyperlipidaemia acts as a major risk factor for CAD and its complications, which related to increased serum levels of total cholesterol (TC) and triglyceride (TG)^4^ and which is a highly hereditary disease and 40%‐60% of variation in blood lipid spectrums was genetically determined.^5^, ^6^ Several compelling researches showed that comprehensive lowering TC,^7^ TG^7^ and low‐density lipoprotein cholesterol (LDL‐C)^8^ levels were more effective in reducing cardiovascular risk than lowering LDL‐C levels alone.^9^ Meanwhile, PCSK9 inhibitors are recommended as class I drugs to further reduce the risk of cardiovascular events in the acute phase of patients suffering from acute coronary syndrome (ACS) by 2019 ESC/EAS guidelines for the management of dyslipidaemias: Lipid modification to reduce cardiovascular risk.^10^ Hence, identification of new lipid‐related genes is important for guiding the treatment of hyperlipidaemia to further reducing cardiovascular risk. Recently, several persuasive genes associated closely with blood lipid levels including the spectrin repeat containing nuclear envelope protein 1 (*SYNE1*) and KH domain containing RNA binding (*QK1*) have been reported by genome‐wide association studies (GWASes) in the European population.^11^ *SYNE1* (also known as dJ45H2.2; 8B; CPG2; AMCM; C6orf98; SCAR8; KASH1; EDMD4; ARCA1; MYNE1; Nesp1, gene ID: 23345, HGNC: 17089, OMIM: 608441) is positioned on chromosome 6q25.2 (Exon count: 154) and encodes a spectrin repeat containing protein expressed in various cell types. Several studies have shown that the *SYNE1* was powerfully related to the metabolism of serum TG, LDL‐C and high‐density lipoprotein cholesterol (HDL‐C) levels.^12^, ^13^, ^14^ Meanwhile, Sharma *et al* also documented that *SYNE1* was related with inferior levels of apolipoprotein (Apo) A1 as well as HDL‐C in severe septic patients.^15^ *QK1* (also known as QK; Hqk; QK1; QK3; hqkI, gene ID: 9444, HGNC: 21100, OMIM: 609590) is positioned on chromosome 6q26 (Exon count: 8) and encodes an RNA binding protein that regulates various cytological functions comprising nuclear mRNA output, mRNA stability, protein translation etc. Previous studies have shown that RNA binding proteins could participate in lipid metabolism by regulating the expression level of lipid‐related genes.^16^

China is a country with multiple ethnicities that are composed of the Han nationality and 55 ethnic minorities. The sixth national census statistics of China (2010) showed that the total population of the Maonan ethnic group was 107 166 (37th). According to the phylogenetic and principal component analyses in recent years, the genetic relationship between Maonan and other minorities in Guangxi,^17^ especially the Buyi,^18^ is much closer than that between Maonan and Han ethnic group.^19^ The marriage culture in Maonan is relatively conservative. Maonan keep the custom of intra‐ethnic marriages, and intermarriage with other ethnic groups is rare. Thus, there was less heterogeneity about their genetic background in Maonan population, so that it is particularly suitable as a population to explore the genetic variation related to blood lipid. Therefore, the current research was designed (a) to evaluate the correlation of the *SYNE1* (rs2623963, rs7745725, rs1358317) and *QK1* (rs9459317, rs1764053, rs1923608, rs16897566) single nucleotide polymorphisms (SNPs) and blood lipid spectrums in participants with hypercholesterolaemia (HCH) and/or hypertriglyceridaemia (HTG); (b) to assess the connection of their haplotypes with the possibility of HCH/HTG; and (c) to recognize the potential G × G as well as G × E interactions among these variants in the Maonan population.

## 2 MATERIALS AND METHODS

### 2.1 Subjects

A total of 1932 discrete individuals (22‐80 years old) were arbitrarily chosen based on our previously stratified randomized samples. They were farmworkers and resided in Huanjiang Maonan Autonomous County, Guangxi Zhuang Autonomous Region. There were 641 unrelated participants with normal lipid levels, 649 unrelated subjects with HCH (TC > 5.17 mmol/L) and 642 unrelated participants with HTG (TG > 1.70 mmol/L). There was not substantial difference in age distribution (54.29 ± 16.54 vs 55.59 ± 14.13/54.64 ± 14.16) and sex ratio between normal and HCH/HTG groups. Patients suffering from HCH did not have high triglyceride levels, and patients suffering from HTG also did not have high cholesterol levels, all subjects were independent and unrelated individuals. They were basically healthy and none of them had any history of type‐2 diabetes mellitus (T2DM), CAD, ischaemic stroke or myocardial infarction. They were not taking any medicines that could alter the serum lipid levels. Prior to the study, all subjects had signed informed consent. The protocol was authorized by the Ethics Committee of the First Affiliated Hospital, Guangxi Medical University.

### 2.2 Epidemiological analysis

Universally standardized methods and protocols were used to conduct the epidemiological survey.^20^ Using a standard set of questionnaires, detailed lifestyle as well as demographic characteristics were collected. Alcohol consumption was categorized into groups of grams of alcohol per day: 0 (non‐drinker), ≤ 25 and > 25.^21^ Smoking status was categorized into groups of cigarettes per day: 0 (non‐smoker), ≤ 20 and > 20.^22^ The inclusion criteria for smoking and drinking have been described in our previous epidemiological studies.^23^, ^24^ The alcohol information included questions about the number of grams of rice wine, wine, beer or liquor consumed during the preceding 12 months. Current smoking was defined as more than one cigarette per day. Participants who reported having smoked ≥ 100 cigarettes during their lifetime were classified as current smokers if they currently smoked and former smokers if they did not.^23^, ^24^ The blood pressure, body mass index (BMI), height, waist circumference and weight were measured as previous description.^25^

### 2.3 Biochemical assays

A fasting venous blood sample of 5 mL was taken from each subject. Two‐fifths of the sample (2 mL) was used to measure serum lipid levels. The remaining three‐fifths of sample (3 mL) was utilized to extract deoxyribonucleic acid (DNA). The methods of serum ApoA1, LDL‐C, ApoB, TG, HDL‐C and TC measurements were referred to a previous study.^26^ All determinations were finished using an autoanalyser (Type 7170A; Hitachi Ltd., Tokyo, Japan) in the Clinical Science Experiment Center of the First Affiliated Hospital, Guangxi Medical University.^6^, ^27^

### 2.4 SNP selection

Following steps were utilized to select seven SNPs in *SYNE1* and *QK1:* (a) *SYNE1*‐*QK1* cluster was chosen from previous GWASes related to serum lipid levels. (b) Haploview (Broad Institute of MIT and Harvard; version 4.2) was utilized to identify tagging SNPs and the latest version of 1000 Genome Project Database was used to predict the functional SNPs that may associate with lipid metabolism. (c) Complete details of the above SNPs were gathered from NCBI dbSNP Build 132. (d) All selected SNPs had been reported that might be related to serum lipid parameters in recent research^11^ and the minor allele frequency (MAF) was > 1%. (e) Seven SNPs of *SYNE1* rs2623963, rs7745725 and rs1358317; and *QK1* rs9459317, rs1764053, rs1923608 and rs16897566 were chosen by the block‐based method. The plan was implemented by marking the connections of linkage disequilibrium (LD) among SNPs (*r* ^2^ > .8).

### 2.5 DNA amplification and genotyping

Genomic DNA was isolated from white blood cells in blood samples by phenol‐chloroform method.^28^, ^29^ All extracted DNA samples were stored at 4°C until experiment. Genotyping of 7 SNPs was performed by the next‐generation sequencing technology (NGS) at the Center for Human Genetics Research, Shanghai Genesky Bio‐Tech Co. Ltd.^30^ Primer sequences of the 7 SNPs are shown in Table S1.

### 2.6 Diagnostic criteria

The values of serum ApoA1 (1.20‐1.60 g/L), HDL‐C (1.16‐1.42 mmol/L), ApoB (0.80‐1.05 g/L), TC (3.10‐5.17 mmol/L), LDL‐C (2.70‐3.10 mmol/L), the ApoA1/ApoB ratio (1.00‐2.50) and TG (0.56‐1.70 mmol/L) were defined as normal at our Clinical Science Experiment Center. The subjects with TG > 1.70 mmol/L were defined as hypertriglyceridaemia (HTG) and TC > 5.17 mmol/L were defined as hypercholesterolaemia (HCH).^4^ The diagnostic criteria of overweight, obesity and normal weight^31^ and hypertension,^32^ were referred to our previous study.

### 2.7 Statistical analyses

All experimental data were statistically assessed by means of SPSS (version 22.0). Values were manifested as mean ± SD, but the levels of TG were manifested as medians and interquartile ranges. Direct counting was used to determine allele frequency. Independent sample *t* test was implemented to assess the common characteristics between the two groups. Chi‐square test was utilized to determine the genotype distribution among different groups. Hardy‐Weinberg equilibrium (HWE), pairwise LD (measured by *D*′), haplotype frequencies and gene‐gene interactions were analysed using Haploview. Analysis of covariance (ANCOVA) was utilized to examine the correlation among serum lipid parameters and genotypes; and *P* < .0071 (equivalent to *P* < .05 after adjusting for 7 independent assessments by the Bonferroni correction) reflected statistical significance. Unconditional logistic regression analysis was utilized to detect the associations among the haplotypes, genotypes and G × G interactions and the probability of HCH/HTG. Related parameters including smoking, gender, blood pressure, BMI, alcohol consumption, blood glucose and age were adjusted for the statistical evaluation. The best interaction combination among SNP‐SNP, haplotype‐haplotype/environment, gene‐gene exposures were screened by generalized multifactor dimensionality reduction (GMDR).^33^ The degree of cross‐validation consistency was an effective method to identify the finest model among all considered probabilities. The score between 0.50 (representing that the model projects no better than chance) and 1.00 (representing impeccable projection) of cross‐validation consistency was an indicator that precisely calculates the extent of case‐control status. *P* < .05 represented statistical significance.

## 3 RESULTS

### 3.1 Common and biochemical characteristics

Table 1 shows that the levels of ApoB, TG, LDL‐C, TC, waist circumference, systolic blood pressure and the proportion of smokers, blood glucose, BMI, diastolic blood pressure, weight and pulse pressure were greater in HCH and HTG than in control groups (*P* < .05‐0.001). The levels of serum HDL‐C, ApoA1 and the ApoA1/ApoB ratio were less in HCH and HTG than in control groups (*P* < .05‐0.001). There was no any obvious difference in the factors including age distribution, height, sex, alcohol consumption between the HCH/HTG and normal groups.

**Table 1:** Comparison of demographic, lifestyle characteristics and serum lipid levels between the control, HCH and HTG populations in Maonan minority

Characteristic	Control	HCH	HTG	*P*_HCH_	*P*_HTG_
Number	641	649	642	—	—
Male/female	288/353	269/380	278/364	.207	.557
Age (years)	54.29 ± 16.54	55.59 ± 14.13	54.64 ± 14.16	.128	.682
Height (cm)	153.38 ± 7.96	154.17 ± 8.44	154.12 ± 9.21	.082	.123
Weight (kg)	51.08 ± 9.44	55.31 ± 10.33	56.61 ± 11.97	.000	.000
Body mass index (kg/m^2^)	21.65 ± 3.38	23.20 ± 3.62	24.63 ± 2.66	.000	.004
Waist circumference	73.80 ± 8.16	78.51 ± 8.94	80.47 ± 9.44	.000	.000
Smoking, n %					
Non‐smoker	507	486	468		
≤20 cigarettes/day	121	127	113		
>20 cigarettes/day	13	36	61	.003	.000
Alcohol, n %)					
Non‐drinker	533	519	513		
≤25 g/d	57	60	54		
>25 g/d	51	70	75	.202	.081
SBP (mm Hg)	130.08 ± 22.51	135.79 ± 22.03	134.69 ± 22.57	.000	.000
DBP (mm Hg)	80.57 ± 12.00	83.43 ± 11.28	83.20 ± 12.55	.000	.000
PP (mm Hg)	49.51 ± 17.15	52.37 ± 17.93	51.50 ± 17.10	.004	.037
Glu (mmol/L)	5.99 ± 1.31	6.22 ± 1.57	6.25 ± 1.45	.004	.001
TC (mmol/L)	4.31 ± 0.67	5.93 ± 0.80	4.39 ± 0.85	.000	.027
TG (mmol/L)	0.95(0.53)	1.15(0.46)	2.34(1.06)	.000	.000
HDL‐C (mmol/L)	1.64 ± 0.46	1.56 ± 0.49	1.43 ± 0.43	.002	.000
LDL‐C (mmol/L)	2.45 ± 0.55	3.40 ± 0.87	2.83 ± 0.89	.000	.000
ApoA1 (g/L)	1.33 ± 0.32	1.29 ± 0.28	1.24 ± 0.24	.008	.000
ApoB (g/L)	0.76 ± 0.16	1.03 ± 0.18	0.97 ± 0.21	.000	.000
ApoA1/ApoB	1.81 ± 0.55	1.41 ± 0.46	1.47 ± 0.51	.000	.000

Note

The value of triglyceride was presented as median (interquartile range) for not a normal distribution.

Mean ± SD determined by t test. Median (interquartile range) tested by the Wilcoxon‐Mann‐Whitney test. The rate or constituent ratio between the different groups was analysed by the chi‐square test.

Abbreviations: Apo, apolipoprotein; DBP, diastolic blood pressure; Glu, blood glucose; HCH, hypercholesterolaemia; HDL‐C, high‐density lipoprotein cholesterol; HTG, hypertriglyceridaemia; LDL‐C, low‐density lipoprotein cholesterol; PP, pulse pressure; SBP, systolic blood pressure; TC, total cholesterol; TG, triglyceride.

### 3.2 Genotypic and allelic occurrences and the association with serum lipid levels

**FIGURE 1:**
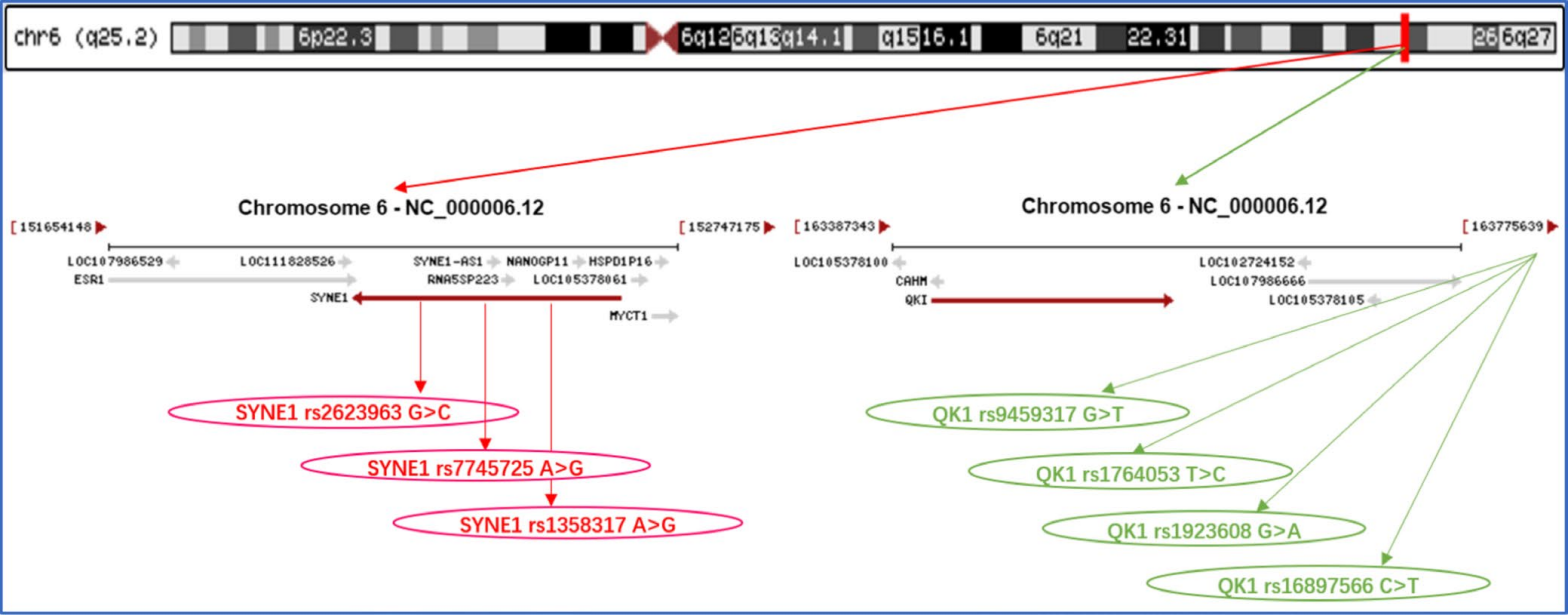
The positions of the *SYNE1* and *QK1* mutations

**FIGURE 2:**
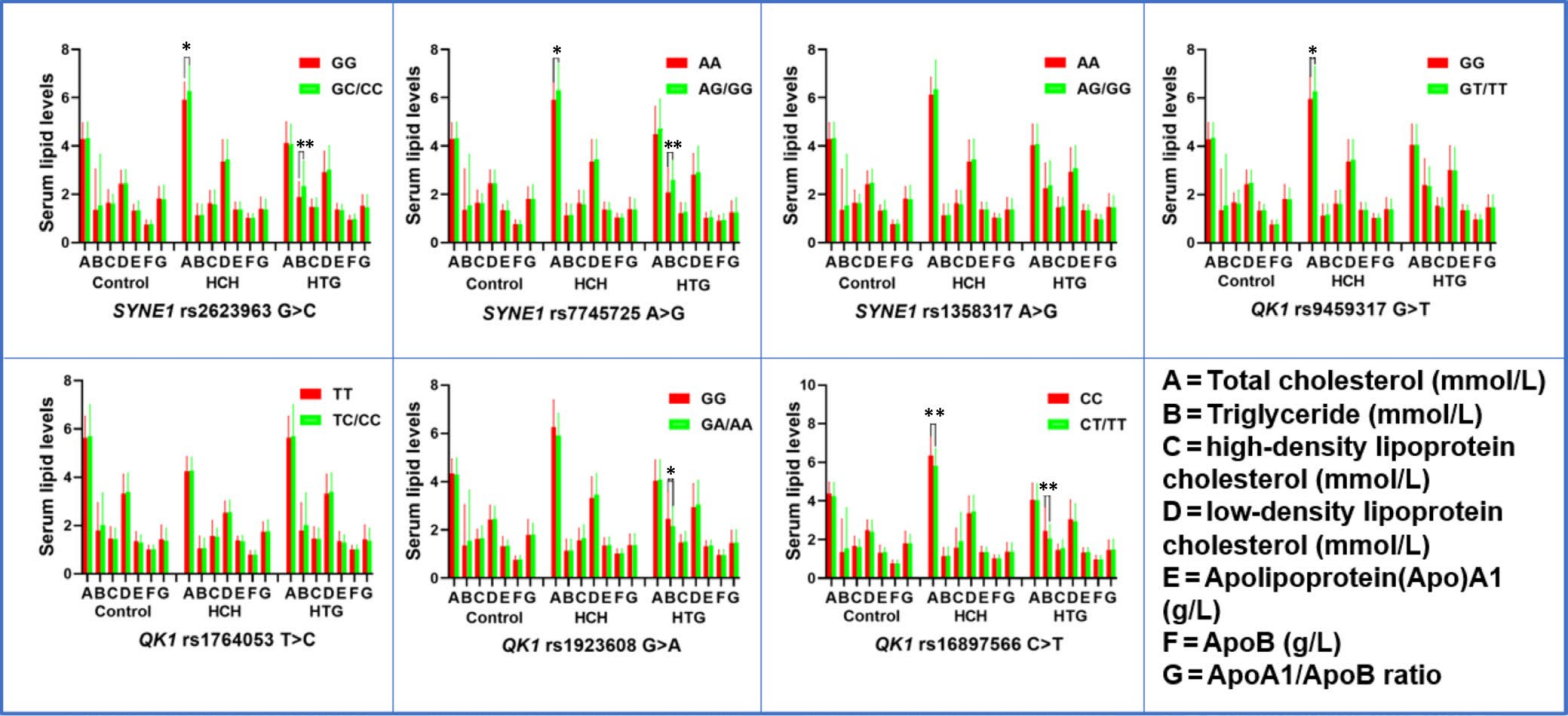
Association between the genotypes of *SYNE1* and *QK1* SNPs and blood lipid levels in the control, HCH and HTG groups. TC, total cholesterol; TG, triglyceride; HDL‐C, high‐density lipoprotein cholesterol; LDL‐C, low‐density lipoprotein cholesterol; Apo, apolipoprotein. ^*^ *P* < .007; ^**^ *P* < .001 (*P* < .007 was considered statistically significant after adjusting by Bonferroni correction)

Seven SNPs of *SYNE1‐QK1* cluster were detected in a close genomic area of chromosome 6 (Figure 1). The genotypic as well as allelic occurrences of the *SYNE1* (rs2623963, rs7745725 and rs1358317) and *QK1* (rs9459317, rs1764053, rs1923608 and rs16897566) SNPs are represented in Table 2. The genotype distribution of 7 SNPs met the Hardy‐Weinberg equilibrium in HCH, HTG and control groups. The genotypic frequencies of following SNPs were suggestively distinctive between the control and HCH groups (rs2623963, rs7745725, rs9459317, rs16897566), or between the control and HTG groups (rs2623963, rs1358317, rs7745725, rs1923608, rs16897566 SNPs; *P* < .05). The dominant models of rs2623963, rs7745725 and rs9459317 SNPs showed an increased morbidity of HCH, whereas the dominant model of rs16897566 revealed a protective effect (*P* < .05). At the same time, the dominant models of rs2623963 and rs7745725 SNPs showed an increased morbidity of HTG, while the dominant models of rs1923608 and rs16897566 indicated a protective effect. As shown in Figure 2, the correlation between the *SYNE1* and *QK1* SNPs and serum lipid parameters including TC (rs2623963 and rs7745725, rs9459317, rs16897566) in subjects with HCH; and TG (rs2623963 and rs7745725, rs1923608, rs16897566) in subjects with HTG were observed (*P* < .007‐0.001).

**Table 2:** The association between the *SYNE1*, *QK1* polymorphisms with hyperlipidaemia [n (%)]

SNP/Genotype	Control (n = 641)	HCH (n = 649)	HTG (n = 642)	* *P*_HCH_	OR (95%CI)_HCH_	*P*_HCH_ ^#^	*P*_HTG_ *	OR (95%CI)_HTG_	^#^ *P*_HTG_
*SYNE1* rs2623963 G > C									
GG	340 (53.0)	300 (46.2)	296 (46.1)		1	‐		1	‐
GC + CC	301 (47.0)	349 (53.8)	346 (53.9)	.014	1.28 (1.01‐1.62)	.041	.013	1.26 (1.00‐1.59)	.046
MAF	338 (26.0）	416 (32.0）	414 (32.0)	.002			0.001		
*P*_HWE_	0.15	0.93	0.86						
*SYNE1* rs7745725 A > G									
AA	364 (56.8）	315 (48.5）	325 (50.6)		1	‐		1	‐
AG + GG	277 (43.2）	334 (51.5）	317 (49.4)	.003	1.38 (1.09‐1.74)	.008	.027	1.22 (0.96‐1.55)	.011
MAF	307 (24.0）	408 (31.0）	382 (30.0)	2.16E‐5			.001		
*P*_HWE_	0.16	0.083	0.13						
*SYNE1* rs1358317 A > G									
AA	369 (57.6）	351 (54.1）	332 (51.7)		1	‐		1	‐
AG + GG	272 (42.4）	298 (45.9）	310 (48.3)	.210	1.10 (0.87‐1.39)	.440	.035	1.19 (0.94‐1.51)	.150
MAF	304 (24.0）	346 (27.0）	373 (29.0)	.085					
*P*_HWE_	0.44	0.69	0.10						
*QK1* rs9459317 G > T									
GG	363 (56.6）	318 (49.0）	328 (53.7)		1	‐		1	‐
GT + TT	278 (43.4）	331 (51.0）	314 (46.3)	.006	1.40 (1.11‐1.78)	.005	.300	1.15 (0.91‐1.46)	.250
MAF	312 (24.0）	401 (31.0）	344 (27.0)	1.96E‐4			.154		
*P*_HWE_	0.45	0.14	0.84						
*QK1* rs1764053 T > C									
TT	226 (35.3）	230 (35.4）	236 (36.8)		1	‐		1	‐
TC + CC	415 (64.7)	419 (64.6)	406 (63.2)	.950	1.03 (0.81‐1.32)	.790	.570	1.03 (0.80‐1.32)	.820
*MAF*	683 (37.0)	620 (42.0)	488 (38.0)	.483			.108		
*P*_HWE_	0.57	0.13	0.079						
*QK1* rs1923608 G > A									
GG	280 (43.7)	310 (47.8)	329 (51.2)		1	‐		1	‐
GA + AA	361 (56.3)	339 (52.2)	313 (48.8)	.141	0.84(0.67‐1.07)	.160	.007	0.76 (0.60‐0.95)	.018
*MAF*	447 (35.0)	395 (30.0)	357 (28.0)	.016			1.15E‐4		
*P*_HWE_	0.16	0.52	0.33						
*QK1* rs16897566 C > T									
CC	311 (48.5)	361 (55.6)	359 (55.9)		1	‐		1	‐
CT + TT	330 (51.5)	288 (44.4)	283 (44.1)	.011	0.75 (0.59‐0.94)	.015	.008	0.78(0.62‐0.91)	.043
MAF	396 (31.0)	325 (25.0)	342 (27.0)	.001			2.70E‐4		
*P*_HWE_	0.40	0.53	1.00						

Note

HCH, hypercholesterolaemia; HTG, hypertriglyceridaemia; HWE, Hardy‐Weinberg equilibrium; MAF, minor allele frequency; *QK1*, the KH domain containing RNA binding gene; *SYNE1*, the spectrin repeat containing nuclear envelope protein 1 gene.

* *P*‐value defined as chi‐square test probability.

^#^ *P*‐value defined as logistic test probability.

### 3.3 Haplotypes and serum lipid levels

**FIGURE 3:**
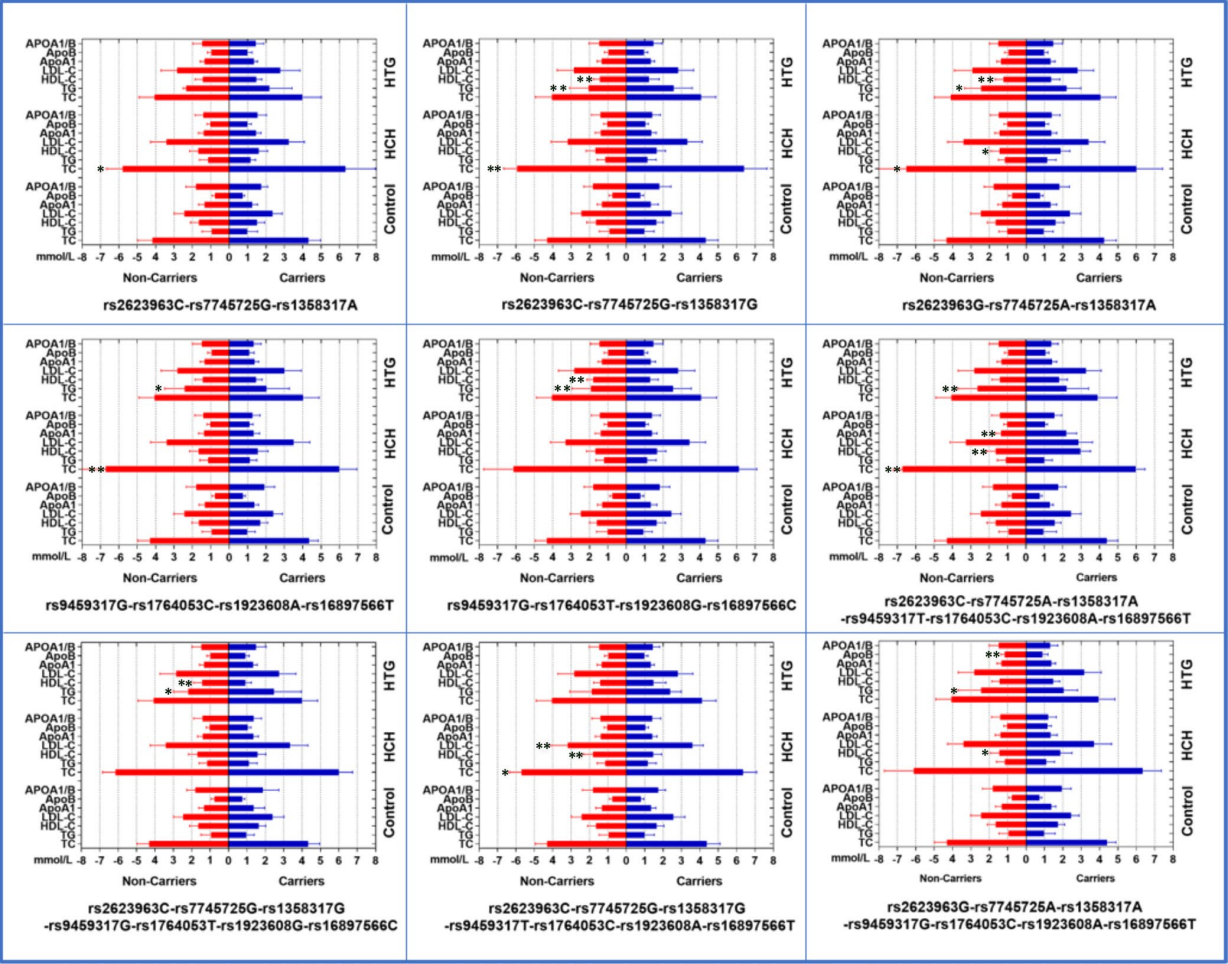
Serum lipid levels according to the haplotypes in the control, HCH and HTG groups. TC, total cholesterol; TG, triglyceride; HDL‐C, high‐density lipoprotein cholesterol; LDL‐C, low‐density lipoprotein cholesterol; Apo, apolipoprotein; ApoA1/B, the ratio of ApoA1 to ApoB. *P < .006 and **P < .001 (*P* < .006 was considered statistically significant after Bonferroni correction)

Figure 3 reveals the effects of nine haplotypes on serum lipid levels. Possible integrative haplotypes or gene‐gene interactions among the detected SNPs were correlated with TC (*SYNE1* C‐G‐A, C‐G‐G, G‐A‐A; *QK1* G‐C‐A‐T; *SYNE1‐QK1* C‐A‐A‐T‐C‐A‐T, C‐G‐G‐T‐C‐A‐T), HDL‐C (*SYNE1* G‐A‐A; *SYNE1‐QK1* C‐A‐A‐T‐C‐A‐T, C‐G‐G‐T‐C‐A‐T, G‐A‐A‐G‐C‐A‐T), ApoA1 (*SYNE1‐QK1* C‐A‐A‐T‐C‐A‐T), and LDL‐C (*SYNE1‐QK1* C‐G‐G‐T‐C‐A‐T) in HCH group, and TG (*SYNE1* C‐G‐G, G‐A‐A; *QK1* G‐C‐A‐T; G‐T‐G‐C, *SYNE1‐QK1* C‐A‐A‐T‐C‐A‐T, C‐G‐G‐G‐T‐G‐C, G‐A‐A‐G‐C‐A‐T), HDL‐C (*SYNE1* C‐G‐G, G‐A‐A; *QK1* G‐T‐G‐C, *SYNE1‐QK1* C‐G‐G‐G‐T‐G‐C), and ApoB (*SYNE1‐QK1* G‐A‐A‐G‐C‐A‐T) in HTG group.

### 3.4 Haplotype‐based association with HCH/HTG

**FIGURE 4:**
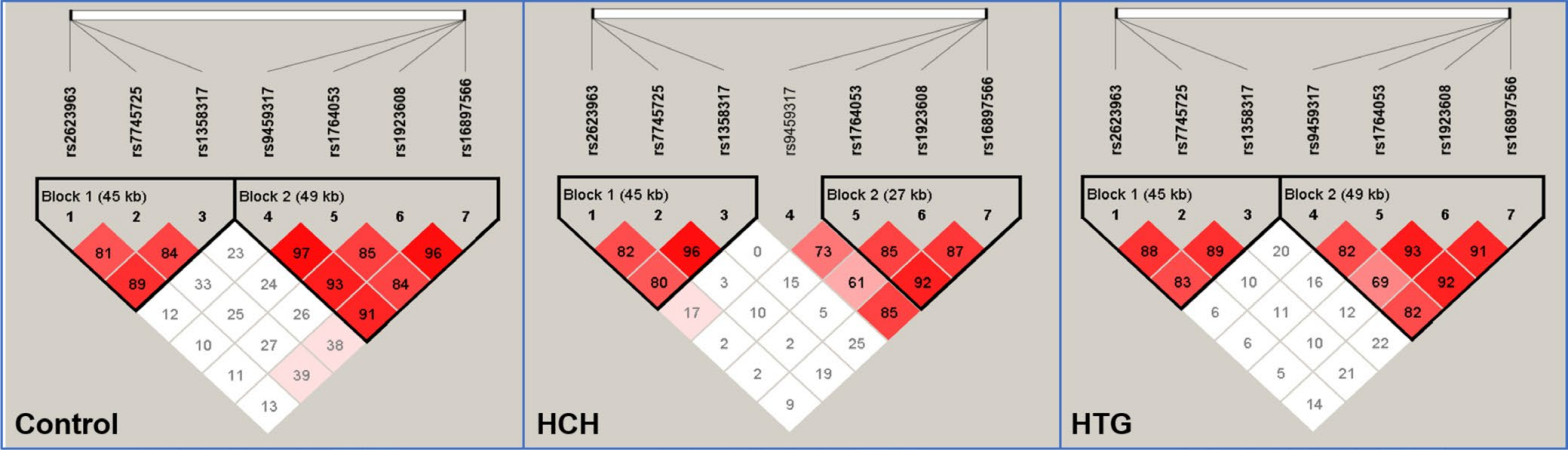
The linkage disequilibrium (LD) represents pairwise *D*′ in the control, HCH and HTG groups

As shown in Figure 4, there was a strong pairwise LD among the detected loci in control, HCH as well as HTG groups. As shown in Table 3, the dominant haplotypes were the *SYNE1* G‐A‐A (> 60% of the samples) and *QK1* G‐T‐G‐C (>50% of the samples). The haplotypes of the *SYNE1* C‐G‐A and C‐G‐G were related to an augmented morbidity of HCH, while the haplotypes of the *SYNE1* G‐A‐A and *QK1* G‐C‐A‐T played a protective role. The haplotypes of the *SYNE1* C‐G‐G and *QK1* G‐T‐G‐C were related to an augmented morbidity of HTG, while the haplotypes of the *SYNE1* G‐A‐A and *QK1* G‐C‐A‐T played a protective role.

**Table 3:** Haplotype frequencies among 3 SNPs of the *SYNE1* and 4 SNPs of the *QK1* genes in the control and HCH/HTG groups [n (frequency)]

No.	Haplotype	Control	HCH	OR (95% CI)_HCH_	*P*_HCH_	HTG	OR (95% CI)_HTG_	*P*_HTG_
S1	C‐A‐A	54.96 (0.043)	51.49 (0.040)	0.914 (0.620‐1.349)	.651335	53.15 (0.041)	0.972 (0.661‐1.429)	.883865
S2	C‐G‐A	18.91 (0.015)	49.98 (0.039)	2.655 (1.555‐4.534)	.000210	16.01 (0.012)	0.844 (0.432‐1.649)	.618385
S3	C‐G‐G	264.13 (0.206)	314.53 (0.242)	1.223 (1.014‐1.474)	.034900	322.15 (0.251)	1.311 (1.087‐1.580)	.004545
S4	G‐A‐A	884.69 (0.690)	832.22 (0.641)	0.764 (0.644‐0.908)	.002170	821.35 (0.640)	0.789 (0.661‐0.942)	.008739
Q1	G‐C‐A‐C	50.09 (0.039	56.03 (0.043)	1.156 (0.783‐1.707)	.465169	50.54 (0.039	1.043 (0.700‐1.555)	.835624
Q2	G‐C‐A‐T	64.44 (0.050)	20.23 (0.016)	0.311 (0.187‐0.515)	1.92e‐006	23.52 (0.018)	0.364 (0.225‐0.588)	1.78e‐005
Q3	G‐C‐G‐C	102.47 (0.080)	92.26 (0.071)	0.918 (0.685‐1.231)	.569560	101.29 (0.079)	1.022 (0.767‐1.362)	.882135
Q4	G‐T‐G‐C	710.29 (0.554)	698.30 (0.538)	1.024 (0.871‐1.203)	0.778006	748.44 (0.583)	1.238 (1.051‐1.458)	.010776
Q5	T‐C‐A‐T	294.80 (0.230)	275.42 (0.212)	0.947 (0.785‐1.143)	.573405	260.40 (0.203)	0.887 (0.733‐1.072)	.214404

Note

The haplotypes of S1‐S4 are combined with *SYNE1* rs2623963‐rs7745725‐rs1358317 and Q1‐Q5 are combined with *QK1* rs9459317‐rs1764053‐rs1923608‐rs16897566.

Rare Hap (frequency < 1%) in both populations has been dropped.

Abbreviations: HCH, hypercholesterolaemia; HTG, hypertriglyceridaemia; *QK1*, the KH domain containing RNA binding gene; *SYNE1*, the spectrin repeat containing nuclear envelope protein 1 gene.

### 3.5 Gene‐gene interaction‐based association with HCH as well as HTG

The commonest gene‐gene interaction haplotype was the *SYNE1*‐*QK1* G‐A‐A‐G‐T‐G‐C (>30% of the samples) (Table 4). The haplotypes of *SYNE1*‐*QK1* C‐A‐A‐T‐C‐A‐T and G‐A‐A‐G‐C‐A‐T were relevant to a decreased morbidity of HCH as well as HTG, while the haplotype of C‐G‐G‐T‐C‐A‐T was relevant to an augmented morbidity of HCH as well as HTG. In addition, the haplotype of C‐G‐G‐G‐T‐G‐C also increased the morbidity of HTG.

**Table 4:** Frequencies of GxG interaction haplotypes among 7 SNPs of the *SYNE1*‐*QK1* gene cluster in control and HCH/HTG groups

No.	G × G interaction haplotypes	Control	HCH	OR (95% CI)_HCH_	*P*_HCH_	HTG	OR (95% CI)_HTG_	*P*_HTG_
	A‐B‐C‐D‐E‐F‐G							
H1	C‐A‐A‐T‐C‐A‐T	39.84 (0.031)	20.24 (0.016)	0.545 (0.317‐0.938)	.026236	16.19 (0.013)	0.419 (0.233‐0.751)	.002643
H2	C‐G‐G‐G‐T‐G‐C	164.29 (0.128)	174.58 (0.134)	1.195 (0.946‐1.508)	.134882	191.57 (0.149)	1.281 (1.019‐1.610)	.033856
H3	C‐G‐G‐T‐C‐A‐T	42.82 (0.033)	57.76 (0.044)	1.502 (1.001‐2.252)	.047878	62.00 (0.048)	1.558 (1.045‐2.323)	.028332
H4	G‐A‐A‐G‐C‐A‐T	52.22 (0.041)	16.98 (0.013)	0.344 (0.197‐0.598)	8.16e‐005	20.43 (0.016)	0.400 (0.238‐0.672)	.000356
H5	G‐A‐A‐G‐C‐G‐C	72.88 (0.057)	68.71 (0.053)	1.031 (0.733‐1.451)	.859732	73.65 (0.057)	1.069 (0.764‐1.496)	.697098
H6	G‐A‐A‐G‐T‐G‐C	501.36 (0.391)	465.25 (0.358)	1.025 (0.861‐1.219)	.784008	482.68 (0.376)	1.026 (0.864‐1.218)	.769440
H7	G‐A‐A‐T‐C‐A‐T	198.21 (0.155)	178.22 (0.137)	0.977 (0.781‐1.223)	.841333	171.24 (0.133)	0.891 (0.712‐1.117)	.317073

Note

Rare Hap (frequency < 1%) in both populations has been dropped.

Abbreviations: A, *SYNE1* rs2623963 G > C; B, *SYNE1* rs7745725 A > G; C, *SYNE1* rs1358317 A > G; D, *QK1* rs9459317 G > T; E, *QK1* rs1764053 T > C; F, *QK1* rs1923608 G > A; G, *QK1* rs16897566 C > T; HCH, hypercholesterolaemia; HTG, hypertriglyceridaemia; *QK1*, the KH domain containing RNA binding gene; *SYNE1*, the spectrin repeat containing nuclear envelope protein 1 gene.

### 3.6 Gene‐gene/environment interaction on hyperlipidaemia

GMDR was utilized to evaluate the association between the G × G/ G × E factor (comprising blood pressure (BP), age, drinking, BMI, glucose, smoking and sex) interactions and the risk of hyperlipidaemia, after adjusting for covariates. A significant three‐locus model (a cross‐validation constancy of 9 of 10, the assessment accurateness of 66.69%, ^#^ *P* < .001) comprising rs7745725, rs9459317 and rs16897566 SNPs was noticed in HCH group and another significant three‐locus model (a cross‐validation constancy of 9 of 10, the assessment accurateness of 58.53%, ^#^ *P* = .006) comprising rs2623963, rs1923608 and rs16897566 SNPs was noticed in HTG group (Table 5, representing a possible SNP‐SNP interaction among the above SNPs). In addition, other significant models including the haplotype‐haplotype (*SYNE1* C‐G‐G, G‐A‐A and *QK1* G‐C‐A‐T) and haplotype‐environment (*SYNE1* C‐A‐A, C‐G‐A and drinking), gene‐gene (*SYNE1‐QK1* C‐A‐A‐T‐C‐A‐T, C‐G‐G‐G‐T‐G‐C and C‐G‐G‐T‐C‐A‐T) interactions were detected in the HCH group. At the same time, other significant models including the haplotype‐haplotype (*QK1* G‐C‐A‐C, G‐C‐G‐C and G‐T‐G‐C) and haplotype‐environment (*SYNE1* C‐A‐A, C‐G‐A and drinking), gene‐gene (*SYNE1‐QK1* C‐A‐A‐T‐C‐A‐T, G‐A‐A‐G‐C‐A‐T and G‐A‐A‐G‐T‐G‐C) interactions were detected in the HTG group.

**Table 5:** GMDR analysis revealed different interactions among SNPs, haplotype, gene and environment

Locus no.	Best combination	Training Bal.Acc	Testing Bal.Acc	Cross‐validation consistency	*P*	*P* ^#^
HCH						
SNP‐SNP interaction						
2	rs7745725 rs9459317	0.6581	0.6204	8/10	.0010	<.001
3	rs7745725 rs9459317 rs16897566	0.6750	0.6669	9/10	.0010	<.001
Haplotype‐haplotype interaction						
2	S3 S4	0.6332	0.6330	10/10	.0010	<.001
3	S3 S4 Q2	0.6505	0.6501	10/10	.0010	<.001
Haplotype‐environment interaction						
2	S1 S2	0.6342	0.6110	6/10	.0010	<.001
3	S1 S2 Drinking	0.6588	0.6500	8/10	.0010	<.001
Gene‐gene interaction						
2	H1 H3	0.5377	0.5382	8/10	.0547	.0421
3	H1 H2 H3	0.5719	0.5761	10/10	.0010	<.001
HTG						
SNP‐SNP interaction						
2	rs2623963 rs16897566	0.5592	0.5423	7/10	.0547	.0421
3	rs2623963 rs1923608 rs16897566	0.5760	0.5853	9/10	.0107	.0060
Haplotype‐haplotype interaction						
2	Q1 Q3	0.5872	0.5697	8/10	.0010	<.001
3	Q1 Q3 Q4	0.6202	0.6165	10/10	.0010	<.001
Haplotype‐environment interaction						
2	S1 S2	0.7146	0.7151	10/10	.0010	<.001
3	S1 S2 Drinking	0.7380	0.7383	10/10	.0010	<.001
Gene‐gene interaction						
2	H1 H4	0.6375	0.6373	10/10	.0010	<.001
3	H1 H4 H6	0.6769	0.6709	10/10	.0010	<.001

Note

The haplotype is combined with *SYNE1* rs2623963‐rs7745725‐rs1358317 and *QK1* rs9459317‐rs1764053‐rs1923608‐rs16897566.

^#^ Indicates 1000 permutation tests.

**FIGURE 5:**
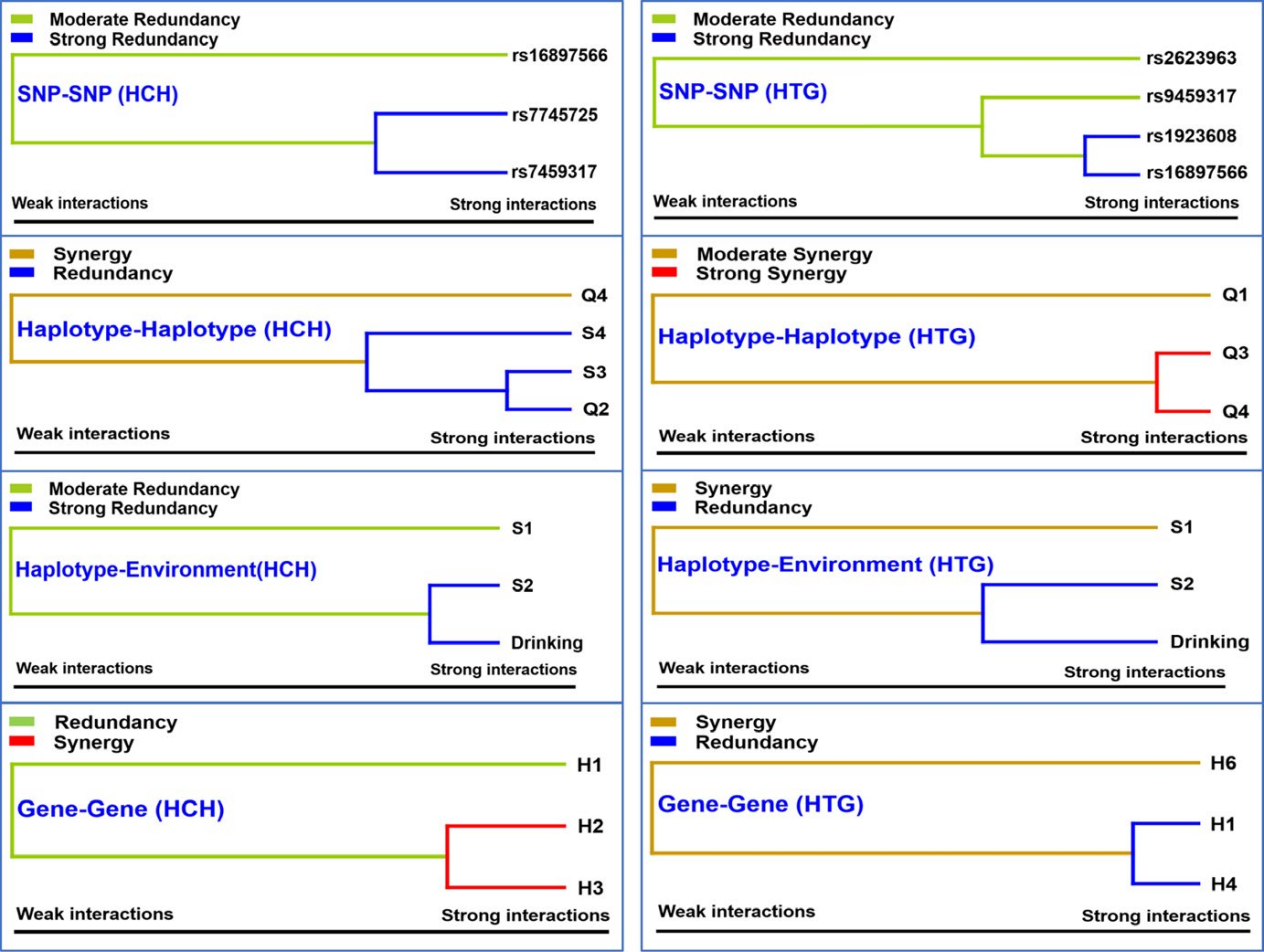
Different types of interaction dendrogram. The strongly interacting elements appear close together at the leaves of the tree, and the weakly interacting elements appear distant from each other

The most powerful synergy was the *SYNE1‐QK1* C‐G‐G‐G‐T‐G‐C and C‐G‐G‐T‐C‐A‐T (gene‐gene) interaction in HCH group and *QK1* G‐C‐G‐C and G‐T‐G‐C (haplotype‐haplotype) interaction in HTG group (Figure 5). Several redundancy interactions including rs7745725 and rs7459317 (SNP‐SNP), *SYNE1* C‐G‐G and *QK1* G‐C‐A‐T (haplotype‐haplotype), *SYNE1* C‐G‐A and drinking (haplotype‐environment) were detected in HCH group, and other redundancy interactions including rs1923608 and rs16897566 (SNP‐SNP), *SYNE1* C‐G‐A and drinking (haplotype‐environment), *SYNE1‐QK1* C‐A‐A‐T‐C‐A‐T and G‐A‐A‐G‐C‐A‐T (gene‐gene) were also detected in HTG group.

The 95% confidence interval (CI) and odds ratio (OR) for the interactions determined by unconditional logistic regression analyses are shown in Table 6. The participants with the genotypes of rs7745725 AG/GG and rs9459317 GT/TT had greater risk of HCH than the ones with the rs7745725 AA and rs9459317 GG genotypes, and the participants with the genotypes of rs1923608 GA/AA and rs16897566 CT/TT had lower risk of HTG than those with the rs1923608 GG and rs16897566 CC genotypes, respectively. The carriers of the *SYNE1* C‐G‐G and *QK1* G‐C‐A‐T decreased the risk of HCH, but the carriers of *SYNE1* C‐G‐A and drinking, and *SYNE1‐QK1* C‐G‐G‐G‐T‐G‐C and C‐G‐G‐T‐C‐A‐T augmented the risk of HCH. Meanwhile, the carriers of the *QK1* G‐C‐G‐C and G‐T‐G‐C increased the risk of HTG, but the carriers of *SYNE1* C‐G‐A and drinking, and *SYNE1‐QK1* C‐A‐A‐T‐C‐A‐T and G‐A‐A‐G‐C‐A‐T reduced the risk of HTG.

**Table 6:** Analysis for different types of interaction by using logistic regression

Variable 1	Variable 2	OR (95% CI)	*P*
HCH			
SNP‐SNP interaction			
rs7745725	rs9459317		
AA	GG	1	—
AA	GT + TT	1.383 (1.021‐1.872)	.036
AG + GG	GG	1.417 (1.047‐1.917)	.024
AG + GG	GT + TT	1.204 (0.990‐1.609)	1.94E‐4
Haplotype‐haplotype interaction			
S3	Q2		
No‐carriers	No‐carriers	1	—
No‐carriers	Carriers	0.310 (0.155‐0.619)	.001
Carriers	No‐carriers	1.195 (0.952‐1.500)	.125
Carriers	Carriers	0.676 (0.273‐1.274)	4.58E‐6
Haplotype‐environment interaction			
S2	Drinking		
No‐carriers	NO	1	—
No‐carriers	YES	0.961 (0.646‐1.381)	.114
Carriers	NO	2.119 (1.126‐3.986)	.020
Carriers	YES	1.766 (0.820‐2.427)	4.85E‐6
Gene‐gene interaction			
H2	H3		
No‐carriers	No‐carriers	1	—
No‐carriers	Carriers	1.183 (0.793‐1.480)	.116
Carriers	No‐carriers	1.218 (0.836‐1.609)	.013
Carriers	Carriers	1.762 (1.112‐3.058)	4.52E‐5
HTG			
SNP‐SNP interaction			
rs1923608	rs16897566		
GG	CC	1	—
GG	CT + TT	0.894 (0.677‐1.118)	.134
GA + AA	CC	0.721 (0.573‐0.907)	.017
GA + AA	CT + TT	0.817 (0.608‐0.921)	.005
Haplotype‐haplotype interaction			
Q3	Q4		
No‐carriers	No‐carriers	1	—
No‐carriers	Carriers	1.296 (0.950‐1.708)	.011
Carriers	No‐carriers	1.113 (0.843‐1.238)	.214
Carriers	Carriers	1.633 (1.353‐2.057)	6.12E‐5
Haplotype‐environment interaction			
S2	Drinking		
No‐carriers	NO	1	—
No‐carriers	YES	0.969 (0.663‐1.208)	.369
Carriers	NO	0.726 (0.335‐0.959)	.133
Carriers	YES	0.838 (0.609‐1.067)	.035
Gene‐gene interaction			
H1	H4		
No‐carriers	No‐carriers	1	—
No‐carriers	Carriers	0.519 (0.294‐0.819)	.024
Carriers	No‐carriers	0.781 (0.562‐1.176)	.154
Carriers	Carriers	0.649 (0.359‐0.901)	3.32E‐4

Note

The haplotype is combined with *SYNE1* rs2623963‐rs7745725‐rs1358317 and *QK1* rs9459317‐rs1764053‐rs1923608‐rs16897566.

### 3.7 Relationship among lipid parameters and alleles or genotypes

**FIGURE 6:**
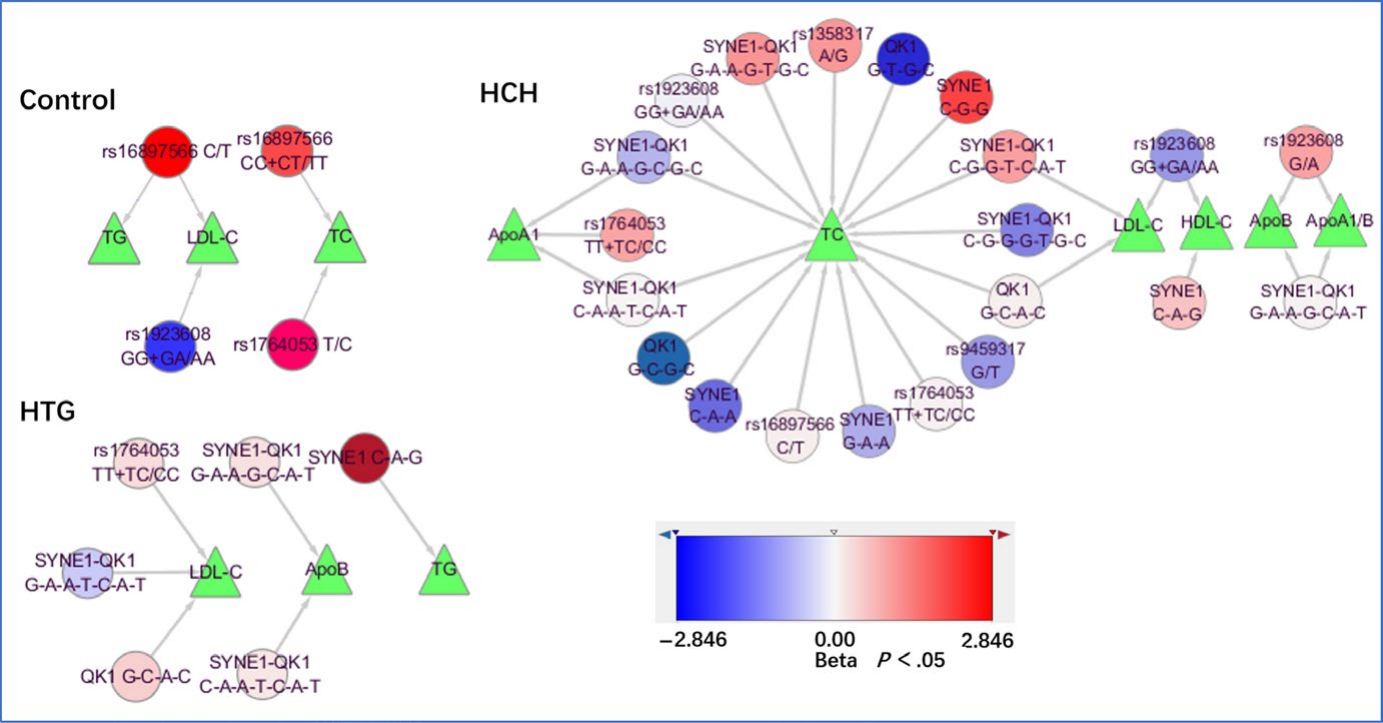
Association of integrative *SYNE1* and *QK1* mutations, haplotypes and G × G interactions with lipid‐related traits in the control, HCT and HCG populations. HDL‐C, high‐density lipoprotein cholesterol; LDL‐C, low‐density lipoprotein cholesterol; Apo, apolipoprotein; HCH, hypercholesterolaemia; HTG, hypertriglyceridaemia; *SYNE1*, the spectrin repeat containing nuclear envelope protein 1 gene; *QK1*, the KH domain containing RNA binding gene

Figure 6 indicates the correlations between alleles and/or genotypes of the 7 SNPs and lipid profiles in the control and HCH and HTG groups. The correlations were evaluated by multivariable linear regression analyses and adjusting for alcohol use, exercise, sex, age, BMI and smoking status. More details are shown in Table S2.

### 3.8 Relative factors for serum lipid parameters

**FIGURE 7:**
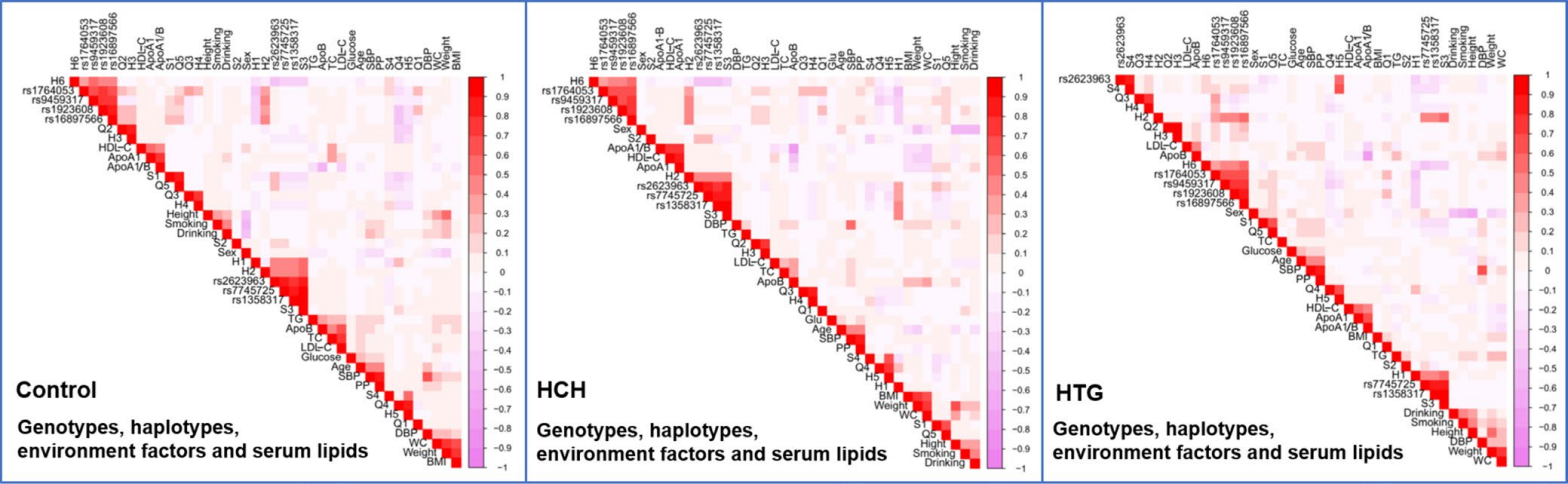
Correlation among environmental exposures, the genotypes and haplotypes of *SYNE1‐QK1* cluster and serum lipid variables in the control, HCH and HTG groups. TC, total cholesterol; TG, triglyceride; HDL‐C, high‐density lipoprotein cholesterol; LDL‐C, low‐density lipoprotein cholesterol; ApoA1, apolipoprotein A1; ApoB, apolipoprotein B; ApoA1/B, the ratio of apolipoprotein A1 to apolipoprotein B; BMI, body mass index, S1‐S4 are combined with *SYNE1* rs2623963‐rs7745725‐rs1358317, Q1‐Q5 are combined with *QK1* rs9459317‐rs1764053‐rs1923608‐rs16897566 and H1‐H7 are combined with *SYNE1* rs2623963‐rs7745725‐rs1358317 and *QK1* rs9459317‐rs1764053‐rs1923608‐rs16897566

As presented in Figure 7, the integrative variants and haplotypes linked with the *SYNE1* rs2623963, rs1358317 and rs7745725 and *QK1* rs9459317, rs1764053, rs1923608 and rs16897566 SNPs to lipid parameters. A series of environmental parameters, for example cigarette smoking, age, alcohol consumption, sex as well as common cardiovascular risk factors just as blood glucose, BP and BMI, were also related with blood lipid levels in control, HCH and HTG groups.

## 4 DISCUSSION

The key outcome of the present research comprised the following aspects: (a) it revealed the correlations of the *SYNE1*‐*QK1* SNPs with blood lipid levels in subjects with HCH as well as HTG. (b) It revealed the frequencies of 7 *SYNE1‐QK1* SNPs, haplotypes and gene‐gene inter‐locus interactions in the Chinese Maonan nationality, which may be a complete complement to the 1000 Genomes database. (c) It provided novel evidence regarding the potential interactions of the *SYNE1‐QK1* SNP‐SNP, haplotype‐haplotype/environment, gene‐gene on blood lipid parameters. (d) It revealed several different gene‐gene (G × G) and gene‐environment (G × E) interactions on the possibility of HCH as well as HTG in the Maonan population.

A lot of studies have shown that hyperlipidaemia, a severe risk factor for CAD, may be due to the combined effects of various elements, for example age, sex, lifestyle, genetic background, environmental factors and their interactions.^34^, ^35^ Previous twin and family genealogy researches have shown that in most populations about 40 to 60 per cent of changes in blood lipid parameters are genetically determined.^36^, ^37^, ^38^ The current research demonstrated the connotation among the *SYNE1‐QK1* SNPs and blood lipid levels. We found that the rs2623963, rs7745725, rs9459317 and rs16897566 SNPs were correlated with serum TC levels in HCH group, and the rs2623963, rs7745725, rs1923608 and rs16897566 SNPs were related with serum TG levels in HTG group. When the correlations of haplotypes and blood lipid levels were analysed, we noticed that the haplotypes of *SYNE1* C‐G‐A, C‐G‐G, G‐A‐A and *QK1* G‐C‐A‐T and *SYNE1‐QK1* C‐A‐A‐T‐C‐A‐T, C‐G‐G‐T‐C‐A‐T were correlated with TC; the haplotypes of *SYNE1* G‐A‐A and *SYNE1‐QK1* C‐A‐A‐T‐C‐A‐T, C‐G‐G‐T‐C‐A‐T, G‐A‐A‐G‐C‐A‐T were related with HDL‐C; the haplotype of *SYNE1‐QK1* C‐A‐A‐T‐C‐A‐T was relevant to ApoA1; and the haplotype of *SYNE1‐QK1* C‐G‐G‐T‐C‐A‐T was correlated with LDL‐C in HCH group. In the meantime, the haplotypes of *SYNE1* C‐G‐G, G‐A‐A and *QK1* G‐C‐A‐T, G‐T‐G‐C and *SYNE1‐QK1* C‐A‐A‐T‐C‐A‐T, C‐G‐G‐G‐T‐G‐C, G‐A‐A‐G‐C‐A‐T were related with TG; the haplotypes of *SYNE1* C‐G‐G, G‐A‐A and *QK1* G‐T‐G‐C and *SYNE1‐QK1* C‐G‐G‐G‐T‐G‐C were related with HDL‐C; and the haplotype of *SYNE1‐QK1* G‐A‐A‐G‐C‐A‐T was correlated with ApoB in HTG group. The correlation analysis based on haplotypes and gene‐gene interactions could illuminate more changes of serum lipids especially for HDL‐C compared to the single SNP alone.

After assessing the correlation of the *SYNE1*‐*QK1* SNPs and the possibility of HCH as well as HTG, the current research revealed that the dominant of rs2623963 and rs7745725 SNPs increased the probability of HCH as well as HTG, whereas the dominant of rs16897566 SNP reduced the possibility of HCH as well as HTG, but the dominant of rs9459317 SNP only increased the risk of HCH and the dominant of rs1923608 SNP only decreased the risk of HTG. The haplotypes of the *SYNE1* C‐G‐A, C‐G‐G and *SYNE1*‐*QK1* C‐G‐G‐T‐C‐A‐T were connected with an augmented morbidity of HCH, the haplotypes of the *SYNE1* C‐G‐G, *QK1* G‐T‐G‐C, *SYNE1*‐*QK1* C‐G‐G‐G‐T‐G‐C, C‐G‐G‐T‐C‐A‐T were correlated with an augmented morbidity of HTG, while the haplotypes of the *SYNE1* G‐A‐A, *QK1* G‐C‐A‐T, *SYNE1*‐*QK1* C‐A‐A‐T‐C‐A‐T, G‐A‐A‐G‐C‐A‐T were correlated with a decreased morbidity of HCH as well as HTG.

GMDR analysis revealed that there were some significant associations with HCH and HTG in two‐ to three‐locus models. The results revealed that there were probable SNP‐SNP (rs7745725, rs9459317 and rs16897566), haplotype‐haplotype (*SYNE1* C‐G‐G, G‐A‐A and *QK1* G‐C‐A‐T), haplotype‐environment (*SYNE1* C‐A‐A, *SYNE1* C‐G‐A and drinking) and gene‐gene (*SYNE1‐QK1* C‐A‐A‐T‐C‐A‐T, C‐G‐G‐G‐T‐G‐C and C‐G‐G‐T‐C‐A‐T) interactions in HCH group. Other potential SNP‐SNP (rs2623963, rs1923608 and rs16897566), haplotype‐haplotype (*QK1* G‐C‐A‐C, G‐C‐G‐C and G‐T‐G‐C), haplotype‐environment (*SYNE1* C‐A‐A, *SYNE1* C‐G‐A and drinking) and gene‐gene (*SYNE1‐QK1* C‐A‐A‐T‐C‐A‐T, G‐A‐A‐G‐C‐A‐T, G‐A‐A‐G‐T‐G‐C) interactions were also noticed in HTG group. Integrated results of GMDR and logistic regression analysis indicated that the participants with the rs7745725 AG/GG and rs9459317 GT/TT genotypes, and the interactions of the *SYNE1* C‐G‐A and drinking, *SYNE1‐QK1* C‐G‐G‐G‐T‐G‐C and C‐G‐G‐T‐C‐A‐T were correlated with an augmented risk of HCH, but the interactions of the *SYNE1* C‐G‐G and *QK1* G‐C‐A‐T reduced the possibility of HCH. Meanwhile, we also revealed that the participants with the rs1923608 GA/AA and rs16897566 CT/TT genotypes, *SYNE1* C‐G‐A and drinking, *SYNE1‐QK1* C‐A‐A‐T‐C‐A‐T and G‐A‐A‐G‐C‐A‐T diminished the probability of HTG, but the interfaces of the *QK1* G‐C‐G‐C and G‐T‐G‐C increased the risk of HTG. Above results indicated that different interaction models between genetic and environmental factors could produce different effects on the onset of HCH and/or HTG. Perhaps a reasonable explanation was that a genetic factor, combined with environmental and lifestyle factors, has been associated with the development of hyperlipidaemia.^39^, ^40^

Maonan ethnic group is famous in China for its unique eating habits. Rice is the staple food for Maonan people. In addition, they also eat corn, potatoes, wheat, sorghum, etc. Maonan people especially prefer the foods of spicy, acid and rich in salt as well as oil. This type of diet rich in long‐term high‐saturated fat might lead to high blood glucose levels, obesity, hyperlipidaemia, hypertension and atherosclerosis.^40^ The main saturated long‐chain fatty acid in the diet could produce harmful effects on serum lipid metabolism, especially impact the levels of serum TC and TG.^41^Unhealthy lifestyle including cigarette smoking and excessive drinking was directly related to the occurrence and development of hyperlipidaemia.^42^ In the current research, we found that the percentages of participants who smoked were greater in HCH and HTG than in control groups. In recent years, the effect of smoking on hyperlipidaemia has gained more and more attention worldwide. Several recent reports have shown that there were lower serum HDL‐C and greater serum TC, LDL‐C and TG levels in smokers compared to non‐smokers.^43^, ^44^ Moderate drinking reduced the incidence of cardiovascular events^45^; and the potential mechanism may be related to increased HDL‐C^46^ and ApoA1^47^ levels. But smoking negated the beneficial effect of drinking on HDL‐C levels. This may explain the difference in serum lipid profiles between both groups. Thus, the combined effects of various eating habits, lifestyle factors as well as environmental aspects perhaps further altered the relationship of hereditary variations and blood lipid levels in the current research.

### 4.1 Study limitations

There are several potential limitations in the current study. Firstly, as compared to many previous large GWASes, the sample size of our study population is a bit small, which might not have enough power to calculate the interaction across the inter‐locus; hence, further researches with a larger sample size are needed to confirm our findings. Secondly, we cannot fully eliminate the influence of dietary habit, lifestyle, physical activity and so on in statistical analysis. Thirdly, patients suffering from both HCH and HTG were not recruited in this research; the genetic information of such patients may be different from those patients suffering from pure HCH or HTG. Consequently, although we have examined the effects of 7 SNPs in the *SYNE1‐QK1* cluster on lipid levels, there are numerous potential lipid‐related SNPs have been overlooked in the current study. In addition, in order to further explore the molecular mechanism of the identified SNPs associated with the development of hyperlipidaemia, several further in‐depth studies including incorporating the genetic information of single nucleotide mutations in *SYNE1‐QK1* cluster, their haplotypes, G × G and G × E interactions in vitro and in vivo functional researches are needed to confirm the impact of a variant on a molecular level.

## 5 CONCLUSIONS

There were potential correlations between the *SYNE1‐QK1*, environment exposures and serum lipid spectrums in the Maonan population. Moreover, the association evaluation based on haplotypes and gene‐gene interactions could improve the power of detecting the risk of dyslipidaemia than anyone of SNP alone, and probably illuminated more changes of serum lipids especially for HDL‐C. When the GMDR was used to analyse the interactions, different interactions between gene and environment factors would produce different redundancy or synergy effects on the morbidity of HCH or HTG. In addition to genetic factors, the influence of environmental exposures on lipid levels would be an important factor that cannot be ignored.

## CONFLICT OF INTEREST

The authors confirm that there are no conflicts of interest.

## AUTHORS’ CONTRIBUTIONS

P.‐F.Z. conceived the study, participated in the design, undertook genotyping, performed the statistical analyses, and drafted the manuscript. R.‐X.Y. conceived the study, participated in the design, carried out the epidemiological survey, collected the samples, and helped to draft the manuscript. C.‐X.L., G.‐X.D., Y.‐Z.G. and B.‐L.W. carried out the epidemiological survey and collected the samples. All authors read and approved the final manuscript.

## Supporting information

Table S1‐S2

## Data Availability

The data sets generated during the present study are not publicly available, because detailed genetic information of each participant was included in these materials.
