# Supplementary material for: SYNE1‐QK1 SNPs, G × G and G × E interactions on the risk of hyperlipidaemia
Source: J Cell Mol Med. 2020 Apr 13;24(10):5772–85. doi: 10.1111/jcmm.15239 (PMC7214174; doi:10.1111/jcmm.15239)
Supplement: Supplementary file 1 — Table S1‐S2 [file JCMM-24-5772-s001.docx]

**Supplementary Table 1** The PCR primers for Next-Generation Sequencing

| **SNP** | **Forward primer** | **Reverse primer** |  |  |  |
| --- | --- | --- | --- | --- | --- |
| ***SYNE1* rs2623963** | AATATACACAGCACACATGTTTCTTTGTC | TGAAAATCGACATCACCATGGGTTT |  |  |  |
| ***SYNE1* rs7745725** | TTGTCACTTCATAGTTGTCTAGGTTATCAG | CCAATTTTCCTTTTTCACAGTTTCACAAAT |  |  |  |
| ***SYNE1* rs1358317** | AACAACCACAGAGGAGTTCCAAATAC | GATCTTGCCCTGAAATGGCAAAA |  |  |  |
| ***QK1* rs9459317** | TCCCAGTTGCATAGAAAAGGTTTTTCT | GGTTCACTGTGCCAAATTGATCTG |  |  |  |
| ***QK1* rs1764053** | TGGTATAAGTCATTCAAAATGAGCTCTGTA | TGAAGCCAAGCTTGGTTTTC |  |  |  |
| ***QK1* rs1923608** | CATAAGGAAGAGAAAAGTGTTTGCGAT | ATTGCTTTTGTGTAGCAAACAGCA |  |  |  |
| ***QK1* rs16897566** | TCCCTTCAGGTCATAAAAATGAG | AGACCAGCTTGTTTCTCACAGAG |  |  |  |

**Supplementary Table 2** Association of integrative SYNE1 and *QK1* mutations, haplotypes and G × G interactions with lipid-related traits in the Control, HCH and HTG populations

| **Lipid** | **SNP** | **Affected**  **allele/**  **Other**  **allele** | **Affected**  **genotype/ Other**  **haplotype** | **Std.error** | **Beta** | **t** | **P** |
| --- | --- | --- | --- | --- | --- | --- | --- |
| **Control** |  |  |  |  |  |  |  |
| TC | *QK1* rs16897566 |  | CC+CT/TT | 0.067 | 0.205 | 3.054 | 0.002 |
|  | *QK1* rs1764053 | T/C |  | 0.070 | -0.144 | -2.060 | 0.040 |
| TG | *QK1* rs16897566 | C/T |  | 0.043 | 0.095 | 2.191 | 0.029 |
| LDL-C | *QK1* rs16897566 | C/T |  | 0.086 | 0.292 | 3.393 | 0.001 |
|  | *QK1* rs1923608 |  | GG+GA/AA | 0.087 | -0.229 | -2.638 | 0.009 |
| **HCH** |  |  |  |  |  |  |  |
| TC | *QK1* rs1358317 | A/G |  | 0.314 | 0.958 | 3.051 | 0.002 |
|  | *QK1* rs1764053 |  | TT+TC/CC | 0.247 | 2.264 | 9.163 | 7.03E-19 |
|  | *QK1* rs1923608 |  | GG+GA/AA | 0.253 | -1.486 | -5.872 | 6.96E-9 |
|  | *QK1* rs16897566 | C/T |  | 0.308 | -1.649 | -5.355 | 1.20E-7 |
|  | *QK1* rs9459317 | G/T |  | 0.331 | -0.976 | -2.948 | 0.003 |
|  | *QK1* G-T-G-C |  | Carriers/Non-carriers | 0.418 | -2.724 | -6.514 | 1.49E-10 |
|  | *SYNE1* C-A-A |  | Carriers/Non-carriers | 0.363 | -1.619 | -4.458 | 9.78E-6 |
|  | *SYNE1-QK1* C-A-A-T-C-A-T |  | Carriers/Non-carriers | 0.508 | 1.781 | 3.508 | 4.83E-4 |
|  | *QK1* G-C-G-C |  | Carriers/Non-carriers | 0.360 | -2.846 | -7.896 | 1.28E-14 |
|  | *SYNE1* C-G-G |  | Carriers/Non-carriers | 0.193 | 1.105 | 5.712 | 1.72E-8 |
|  | *SYNE1-QK1* G-A-A-G-T-G-C |  | Carriers/Non-carriers | 0.373 | 1.164 | 3.125 | 0.002 |
|  | *SYNE1-QK1* C-G-G-T-C-A-T |  | Carriers/Non-carriers | 0.245 | -1.064 | -4.336 | 1.69E-5 |
|  | *QK1* G-C-A-C |  | Carriers/Non-carriers | 0.303 | -1.439 | -4.753 | 2.49E-6 |
|  | *SYNE1-QK1* C-G-G-G-T-G-C |  | Carriers/Non-carriers | 0.244 | -0.910 | -3.725 | 2.13E-4 |
|  | *SYNE1-QK1* G-A-A-G-C-G-C |  | Carriers/Non-carriers | 0.360 | 1.188 | 3.301 | 0.001 |
|  | *SYNE1* G-A-A |  | Carriers/Non-carriers | 0.355 | -0.825 | -2.323 | 0.020 |
| HDL-C | *QK1* rs1923608 |  | GG+GA/AA | 0.038 | 0.092 | 2.397 | 0.017 |
|  | *SYNE1* C-A-G |  | Carriers/Non-carriers | 0.085 | 0.192 | 2.256 | 0.024 |
| LDL-C | *SYNE1-QK1* C-G-G-T-C-A-T |  | Carriers/Non-carriers | 0.092 | 0.255 | 2.785 | 0.006 |
|  | *QK1* rs1923608 |  | GG+GA/AA | 0.123 | -0.371 | -3.007 | 0.003 |
|  | *QK1* G-C-A-C |  | Carriers/Non-carriers | 0.128 | 0.335 | 2.616 | 0.009 |
| ApoA1 | *SYNE1-QK1* C-A-A-T-C-A-T |  | Carriers/Non-carriers | 0.071 | 0.168 | 2.365 | 0.018 |
|  | *QK1* rs1764053 |  | TT+TC/CC | 0.024 | 0.061 | 2.584 | 0.010 |
|  | *SYNE1-QK1* G-A-A-G-C-G-C |  | Carriers/Non-carriers | 0.039 | -0.079 | -2.054 | 0.040 |
| ApoB | *QK1* rs1923608 | G/A |  | 0.025 | -0.079 | -3.153 | 0.002 |
|  | *SYNE1-QK1* G-A-A-G-C-A-T |  | Carriers/Non-carriers | 0.052 | 0.160 | 3.045 | 0.002 |
| ApoA1/B | *QK1* rs1923608 | G/A |  | 0.065 | 0.174 | 2.685 | 0.007 |
|  | *SYNE1-QK1* G-A-A-G-C-A-T |  | Carriers/Non-carriers | 0.135 | -0.276 | -2.047 | 0.041 |
| **HTG** |  |  |  |  |  |  |  |
| TG | SYNE1 C-A-G |  | Carriers/Non-carriers | 0.563 | 1.649 | 2.929 | 0.004 |
| LDL-C | rs1764053 |  | TT+TC/CC | 0.079 | 0.176 | 2.234 | 0.026 |
|  | QK1 G-C-A-C |  | Carriers/Non-carriers | 0.144 | 0.266 | 1.847 | 0.045 |
|  | SYNE1-QK1 G-A-A-T-C-A-T |  | Carriers/Non-carriers | 0.098 | -0.302 | -0.3076 | 0.002 |
| ApoB | SYNE1-QK1 G-A-A-G-C-A-T |  | Carriers/Non-carriers | 0.049 | 0.145 | 2.943 | 0.003 |
|  | SYNE1-QK1 C-A-A-T-C-A-T |  | Carriers/Non-carriers | 0.049 | 0.103 | 2.090 | 0.037 |

*HDL-C*, high density lipoprotein cholesterol; *LDL-C*, low density lipoprotein cholesterol; *Apo*, apolipoprotein; *HCH*, hypercholesterolemia; *HTG*, hypertriglyceridemia; *SYNE1*, the spectrin repeat containing nuclear envelope protein 1 gene; *QK1*, the KH domain containing RNA binding gene.
